# Diabetes Mellitus Aggravates Hemorrhagic Transformation after Ischemic Stroke via Mitochondrial Defects Leading to Endothelial Apoptosis

**DOI:** 10.1371/journal.pone.0103818

**Published:** 2014-08-18

**Authors:** Keisuke Mishiro, Takahiko Imai, Sou Sugitani, Akira Kitashoji, Yukiya Suzuki, Toshinori Takagi, Huayue Chen, Yasunori Oumi, Kazuhiro Tsuruma, Masamitsu Shimazawa, Hideaki Hara

**Affiliations:** 1 Molecular Pharmacology, Department of Biofunctional Evaluation, Gifu Pharmaceutical University, Gifu, Japan; 2 Departments of Neurosurgery, Gifu University Graduate School of Medicine, Gifu, Japan; 3 Department of Anatomy, Gifu University Graduate School of Medicine, Gifu, Japan; 4 Division of Instrument Analysis, Life Science Research Center, Gifu University, Gifu, Japan; National Centre for Scientific Research “Demokritos”, Greece

## Abstract

Diabetes is a crucial risk factor for stroke and is associated with increased frequency and poor prognosis. Although endothelial dysfunction is a known contributor of stroke, the underlying mechanisms have not been elucidated. The aim of this study was to elucidate the mechanism by which chronic hyperglycemia may contribute to the worsened prognosis following stroke, especially focusing on mitochondrial alterations. We examined the effect of hyperglycemia on hemorrhagic transformation at 24 hours after middle cerebral artery occlusion (MCAO) in streptozotocin (STZ) -induced diabetic mice. We also examined the effects of high-glucose exposure for 6 days on cell death, mitochondrial functions and morphology in human brain microvascular endothelial cells (HBMVECs) or human endothelial cells derived from induced pluripotent stem cells (iCell endothelial cells). Hyperglycemia aggravated hemorrhagic transformation, but not infarction following stroke. High-glucose exposure increased apoptosis, capase-3 activity, and release of apoptosis inducing factor (AIF) and cytochrome c in HBMVECs as well as affected mitochondrial functions (decreased cell proliferation, ATP contents, mitochondrial membrane potential, and increased matrix metalloproteinase (MMP)-9 activity, but not reactive oxygen species production). Furthermore, morphological aberration of mitochondria was observed in diabetic cells (a great deal of fragmentation, vacuolation, and cristae disruption). A similar phenomena were seen also in iCell endothelial cells. In conclusion, chronic hyperglycemia aggravated hemorrhagic transformation after stroke through mitochondrial dysfunction and morphological alteration, partially via MMP-9 activation, leading to caspase-dependent apoptosis of endothelial cells of diabetic mice. Mitochondria-targeting therapy may be a clinically innovative therapeutic strategy for diabetic complications in the future.

## Introduction

Diabetes mellitus (DM) is a severe health problem of epidemic proportions, which continues to expand exponentially worldwide: it is projected that 347 million people are affected and it will reach 439 million by the year 2030 [1]–[3]. Stroke is a major complication in DM patients, and DM increases the risk of stroke by 1.5 to 3-fold as compared to the general population [4]–[7]. Several epidemiological studies have suggested that ischemic stroke patients with DM display a distinct risk-factor and etiologic profile as well as a worse vascular prognosis, higher in-hospital mortality, and slower functional recovery than non-DM patients [8], [9]. A chronically high-level of serum glucose may be a key contributor to the poor outcome observed after cerebral ischemia in DM patients [10].

Many factors contribute to the poor prognosis in stroke patients with DM. Importantly, chronic hyperglycemia is associated with hemorrhagic complications in acute ischemic stroke patients who received thrombolytic therapy [11]; this has also been confirmed in animal models [12]–[14]. In addition, many deleterious pathways involved in the aggravation of the cerebrovascular disorder that results from DM have been reported, including oxidative stress [15], impaired leukocyte function [16], abnormal angiogenesis [17], increased blood-brain barrier permeability [18], and other inflammatory responses [19]–[21]. Nevertheless, the mechanisms underlying the adverse effects of chronic hyperglycemia on cerebral blood vessels have not been fully elucidated.

Mitochondria are complex organelles that perform diverse vital functions such as cellular metabolism, growth, differentiation, and homeostasis. In particular, they play a critical role in cell survival and death by regulating ATP synthesis through lipid and glucose metabolism, reactive oxygen species (ROS) generation, calcium homeostasis, apoptosis stimulation, and aging [22], [23]. Therefore, any alterations in these mitochondrial functions can greatly affect cell fate and tissue function, and occasionally accelerate the morbidity in a fatal capacity.

The importance of altered mitochondrial dynamics in DM is being increasingly recognized [24]. Recent works have demonstrated various abnormalities in mitochondrial networks under hyperglycemic conditions in a variety of cell types, including islet cells [25], [26], hepatocytes [27], skeletal muscle cells [28], [29], circulating blood mononuclear cells [30], and retinal or coronary endothelial cells [31], [32]. However, their role in the human cerebrovascular endothelial cells is currently unknown.

The aim of this study was to elucidate the mechanism by which chronic hyperglycemia may contribute to the worsened prognosis following stroke in DM patients. We used human brain microvascular endothelial cells, and investigated the effects of chronic high-glucose exposure on apoptotic cell death, mitochondrial functions, and morphological alterations to clarify the pathophysiological roles of mitochondria in DM. Finally, we examined the effects of chronic high-glucose exposure on highly purified human endothelial cells derived from induced pluripotent stem (iPS) cells which supposed to more stabilized result.

## Materials and Methods

### Animal model

**Figure 1:**
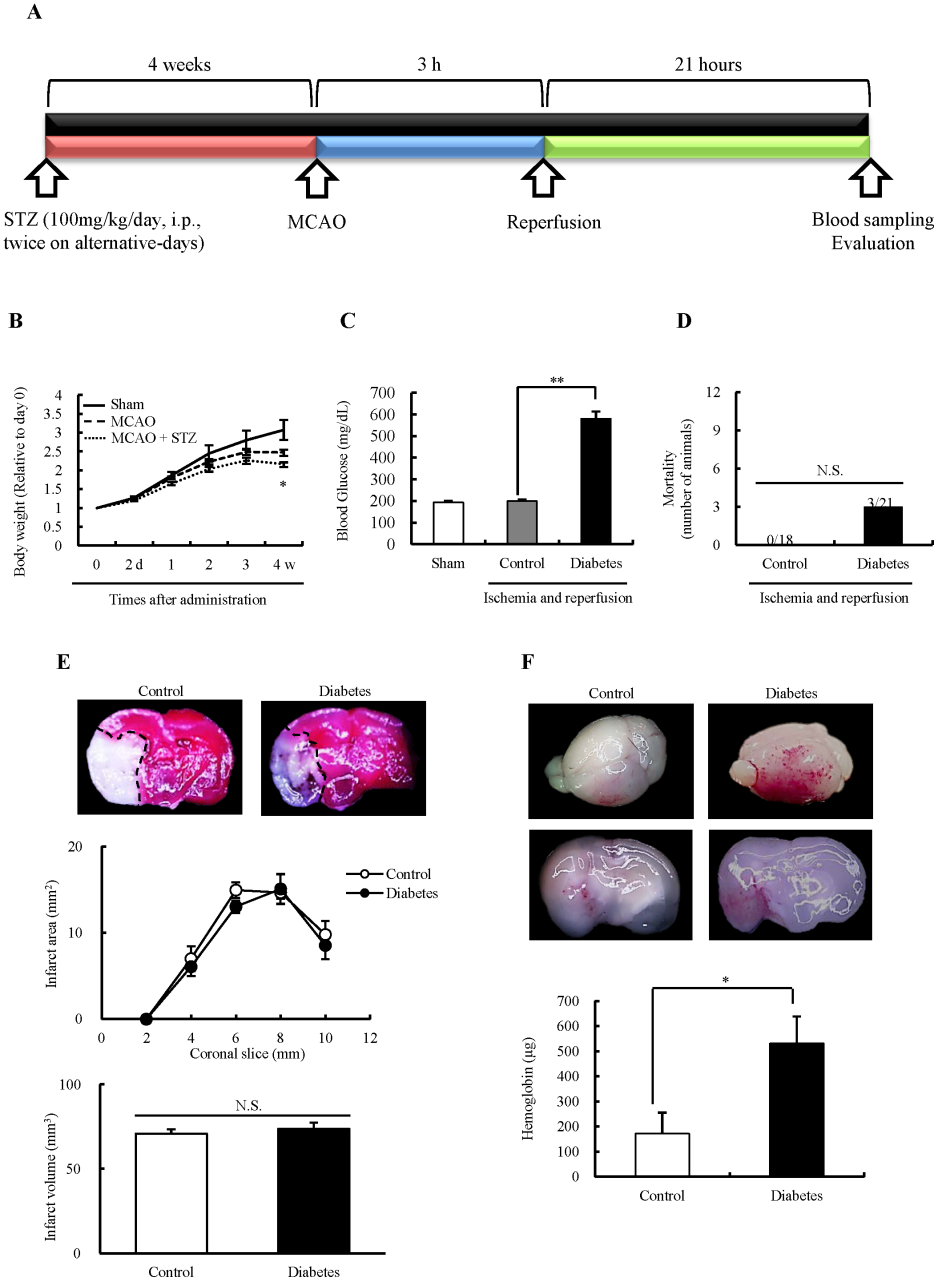
Effects of chronic hyperglycemia on acute ischemic stroke in mice. A: Experimental protocol *in vivo*. A total of 44 mice were evaluated (Sham; n = 5, Control; n = 18, Diabetes; n = 21). STZ, streptozotocin; MCAO, middle cerebral artery occlusion. B: Changes in body weight after reperfusion. *P<0.05 vs. Sham (Student's *t*-test). C: Blood glucose levels at 21 h after reperfusion. **P<0.01 vs. Control (Student's *t*-test). D: Mortality, determined at 21 h after the reperfusion (Chi-square test test). E: Infarct area and volume at 21 h after the reperfusion (Control; n = 7, Diabetes; n = 6). Representative coronal sections were located 1 mm posterior to bregma. TTC-stained coronal sections show infarct tissues (pale unstained region). F: Hemorrhagic volume at 21 h after the reperfusion (Control; n = 11, Diabetes; n = 12). *P<0.05 vs. Control (Student's *t*-test). Representative images of whole brains and coronal sections located in 1 mm posterior to the bregma, respectively. All data are expressed as mean ± SEM.

The experimental designs and all procedures were approved by the Gifu Pharmaceutical University Animal Experimental Committee. All procedures relating to animal care and treatment conformed to the animal care guidelines of this committee. All *in vivo* experiments were performed using male ddY mice (3 weeks old; body weight, 10–20 g; Japan SLC Ltd., Shizuoka, Japan). A model of diabetes was induced by intraperitoneal (i.p.) injection of streptozotocin (STZ) (Wako Pure Chemical Industries, Osaka, Japan) (100 mg/kg/day dissolved in saline) twice on alternative-days. Mice were housed for an additional 4 weeks without insulin supplements. During this period, the vehicle group received i.p. injections of saline, without STZ, under the same dosing regimen (time and volume) used in the STZ-treated group. We monitored body weight for 4 weeks (Fig. 1B). Following the 4-week period, an experimental MCAO model was prepareed as previously described [33]. Briefly, mice were anesthetized with 2.0 to 2.5% isofluorane in 30% oxygen and 70% nitrous oxide via a facemask (Soft Lander; Sin-ei Industry, Saitama, Japan). An 8-0 nylon monofilament (Ethicon, Somerville, NJ, USA) was used via the internal carotid artery to induce focal cerebral ischemia. After 3 h of occlusion, the nylon was gently withdrawn to restore blood flow in the MCA territory and the incision was closed. In all animals, body temperature was maintained between 37.0 and 37.5°C during surgery, with the aid of a heating lamp and heating pad. At 21 h after the reperfusion (just before the sampling), blood samples were collected from the inferior vena cava and serum glucose levels were determined using a glucose detection kit (Glucose CII Test Wako). Animals with blood glucose levels exceeding 300 mg/dL were selected as the “diabetic mice” and were used for subsequent experiments. In this study, we used a total of 61 mice and excluded 17 mice (27.7%) based on the criteria for exclusion, which we set before the experiments. Exclusion criteria were as follows: (a) abnormal serum glucose levels, (b) death due to excessive anesthesia or technical problems during operations, and (c) no infarction in the core area. All experiments were performed in a blinded-manner and randomized for each groups to exclude any bias.

### Infarct volume assessment

At 21 h after the reperfusion, mice were killed by decapitation. Brains were immediately removed and cut into 5 serial 2-mm-thick coronal block slices. These slices were immersed in a 2% solution of 2,3,5-triphenyltetrazolium chloride (TTC) (Sigma-Aldrich, St Louis, MO, USA) for 20 min. Image- J image-processing software was used to measure the unstained areas of the total infarctions, and the infarct volume was calculated as reported previously [34] (Control; n = 7, Diabetes; n = 6).

### Spectrophotometric assay of hemoglobin content

At 21 h after reperfusion, mice were given an overdose of pentobarbital sodium (Dainippon Sumitomo Pharma, Osaka, Japan) and transcardially perfused with cold saline. Brains were immediately removed and cut into 5 serial 2-mm-thick coronal block slices. In our preliminary study, we found that the reproducibly largest infarct size was in the cortical region of the left hemisphere on the anterior fourth slice. Therefore, we analyze the extent of hemorrhagic transformation in this area. After adding 10 ml/g of saline to individual samples, they were homogenized, followed by 30-min of centrifugation (13,000 rpm). A 200 µl volume of reagent (QuantiChrom Hemoglobin Assay Kit; BioAssay Systems, CA, USA) was then added to 50 µl of supernatant. After 15 min, optical density was measured at 400 nm with a spectrophotometer (Skan It RE for Varioskan 133 Flash 2.4; Thermo Fisher Scientific, Waltham, MA, USA). The total hemoglobin content was expressed as micrograms per sample (Control; n = 11, Diabetes; n = 12).

### Cell cultures and incubation under various glucose concentrations

**Figure 2:**
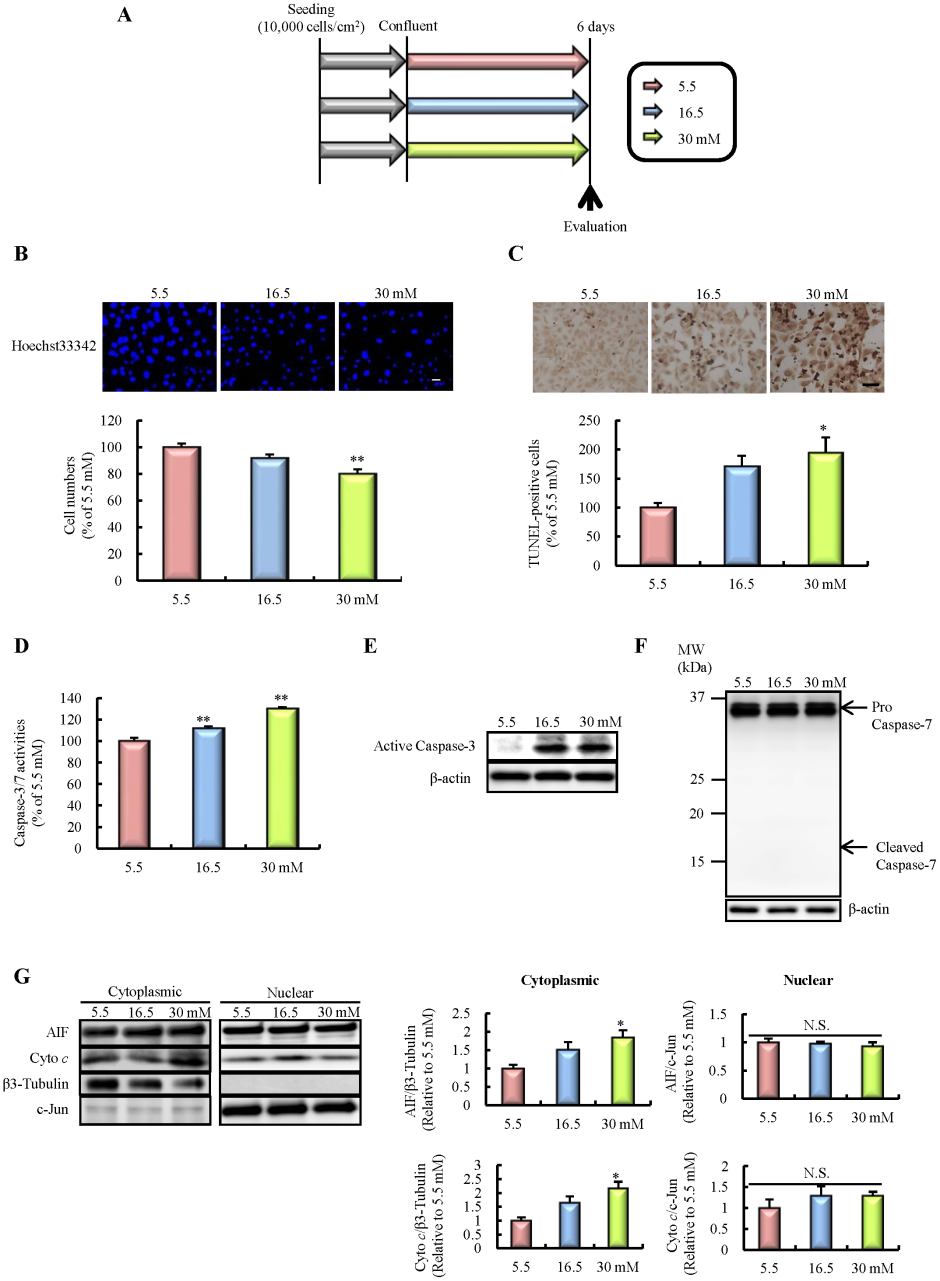
Effects of chronic high-glucose exposure on apoptotic cell death in HBMVECs. A: Experimental protocol *in vitro*. B: Nuclear staining for Hoechst 33342. The number of cells exhibiting nuclear stain was counted (n = 10). The scale bar indicates 50 µm. C: Number of TUNEL-positive cells (n = 4). The scale bar indicates 100 µm. D: Caspase-3/7 activities (n = 10). E: Immunoblotting for Active Caspase-3. F: Immunoblotting for Caspase-7. G: Release of apoptosis inducing factor (AIF) and cytochrome *c* (Cyto *c*) into cytosol, and transit into the nucleus (n = 3). All data are expressed as mean ± SEM (shown as ratio to 5.5 mM). *P<0.05, **P<0.01 vs. 5.5 mM (Dunnet's test). HBMVECs, human brain microvascular endothelial cells.

Human brain microvascular endothelial cells (HBMVECs) (Cell Systems, Kirkland, WA, USA) and iCell endothelial cells (iPS Academia Japan, Inc., Kyoto, Japan) were cultured in 100-mm dishes in HuMedia EG2 (Kurabo, Osaka, Japan), supplemented with 10% heat-inactivated fetal bovine serum (FBS) and VascuLife VEGF Medium (Kurabo) supplemented with specialized iCell Endothelial Cells Medium Supplement, respectively, at 37°C under a humidified 5% CO_2_ atmosphere until they reached 80% confluence. Cells were then transferred into culture plates (1×10^4^ cells/cm^2^) and cultured until they reached confluence. After confluence was achieved, the medium was changed to the conditioned medium containing 5.5 mM (equivalent to the physiological glucose concentration; used as a control), 16.5 mM, or 30 mM (high-glucose exposure) glucose concentrations, and cultivated for an additional 6 days (Fig. 2A). During this period, the culture medium was replaced every 2 days.

### Nuclear staining assays

We evaluated the cell numbers by nuclear staining assays using the fluorescent dye: Hoechst 33342 (Invitrogen, Carlsbad, CA, USA). After high-glucose exposure for 6 days, Hoechst 33342 was added, followed by incubation for 15 min (a final concentration of Hoechst 33342, 8.1 µM). Images were collected using an Olympus IX70 inverted epifluorescence microscope (Olympus, Tokyo, Japan). The total number of cells was counted in a blind manner.

### TUNEL staining

TUNEL staining was performed according to the manufacturer's protocols (In Situ Cell Death Detection kit; Roche Diagnostics, Mannheim, Germany). Briefly, cells were fixed in a freshly prepared 4% paraformaldehyde solution for 1 h, and then blocked in 3% H_2_O_2_ in methanol for 10 min. Cells were further permeabilized in 0.1% Triton X-100 in citric sodium for 2 min on ice. Cells were then incubated with terminal deoxyribonucleotidyl transferase enzyme at 37°C for 1 h, and subsequently incubated with an anti-fluorescein antibody peroxidase conjugate in a humidified chamber for 30 min. Next, the reaction was developed using diaminobenzidine tetrahydrochloride peroxidase substrate. Light microscope images were photographed in five areas per well, and the number of TUNEL-positive cells was counted.

### Caspase 3 and 7 activation measurement

Caspase 3 and 7 was measured by using Caspase-Glo 3/7 Assay (Promega, Madison, WI, USA), according to the manufacturer's instructions. After high-glucose exposure for 6 days, caspase-Glo 3/7 Reagent was added in a 1∶1 ratio of reagent volume to the sample volume. Thereafter, cells were incubated for 1 h at 37°C. The luminescence of each sample was measured using a microplate reader (Varioskan Flash 2.4; Thermo Fisher Scientific).

### Immunoblotting

Cells were lysed in RIPA buffer (Sigma-Aldrich) containing 1% protease inhibitor cocktail and 1% of each phosphatase inhibitor cocktails 2 and 3 (Sigma-Aldrich), and harvested. Lysates were agitated with rotated slowly for 3 h at 4°C. Protein concentrations were measured using a BCA Protein Assay Kit (Thermo Scientific, Rockford, IL, USA) with a bovine serum albumin standard. Thereafter, equal volumes of protein sample and sample buffer were mixed, and the samples were boiled for 5 min at 100°C. The samples (2 µg protein/well) were subjected to 5–20% SDS-PAGE gradient electrophoresis and then transferred to polyvinylidene difluoride membranes (Immobilon-P; Millipore). For immunoblotting, the following primary antibodies were used: mouse anti-Active Caspase-3 antibody (1∶1000 dilution; Genlantis, San Diego, CA, USA), mouse anti-Caspase-7 antibody (1∶1000 dilution; MBL, Nagoya, Japan), mouse anti-AIF antibody (1∶1000 dilution; Santa Cruz, CA, USA), mouse anti-cytochrome c antibody (1∶200 dilution; Santa Cruz), mouse anti-β3-Tubulin antibody (1∶1000 dilution; Cell Signaling Technology, Danvers, MA, USA), rabbit anti-c-Jun antibody (1∶1000 dilution; Cell Signaling Technology), mouse anti-4-hydroxy-2-nonenal antibody (1∶10 dilution: JaICA, Shizuoka, Japan), mouse anti-HSP60 antibody (1∶1000 dilution; StressMarq Biosciences, Victoria, BC, Canada), rabbit anti-VDAC1 antibody (1∶100 dilution; Abgent, Inc., San Diego, CA, USA), and β-actin mouse monoclonal antibody (1∶5000 dilution; Sigma-Aldrich). Horseradish peroxidase (HRP)-conjugated goat anti rabbit antibody (Pierce Biotechnology, Rockford, IL) and HRP-conjugated goat anti mouse antibody were used as secondary antibodies. Immunoreactive bands were visualized using Immunostar-LD (Wako) and a LAS-4000 luminescent image analyzer (Fuji Film Co., Ltd., Tokyo, Japan).

### Subcellular fractionation

To isolate cytosol and nuclear components, we used ProteoExtract Subcellular Proteome Extraction Kit (Calbiochem, San Diego, CA, USA) according to the manufacturer's protocol. Furthermore, to isolate cytosol and mitochondria, we used a Mitochondria Isolation Kit for Cultured Cells (Thermo Fisher Scientific), according to the manufacturer's protocol.

### Cell proliferation assay

Cell proliferation was assessed by culturing cells in a conditioned medium containing 10% WST-8 (Cell Counting Kit-8; Dojin Kagaku, Kumamoto, Japan) for 3 h at 37°C, and then scanning with a microplate reader (Varioskan Flash 2.4; Thermo Fisher Scientific).

### Measurement of cellular ROS production

To evaluate cellular ROS production, we examined the levels of hydrogen peroxide using 5-(and-6)-chloromethyl-2′,7′-dichlorodihydrofluorescein diacetate, acetyl ester (CM-H_2_DCFDA; Invitrogen). Treated cells were incubated with CM-H_2_DCFDA for 1 h at 37°C and then washed with PBS. Fluorescent signals were measured using a Varioskan Flash 2.4 microplate reader (Thermo Fisher Scientific) at 485 nm (excitation) and 535 nm (emission).

### Detection of MMP-2 and MMP-9

We examined MMP-2 and MMP-9 activity using a gelatin zymography kit (Cosmo Bio, Tokyo, Japan). In brief, samples (5 µg protein/well) were loaded onto 10% SDS-polyacrylamide gels containing 1 mg/ml gelatin as substrate. After electrophoresis, gels were incubated in the Wash buffer at room temperature for 1 h with gentle shaking. The gel was further incubated in enzyme reaction buffer at 37°C for 40 h. After incubation, the gel was immersed in staining solution for 30 min, followed by de-staining solution for 3 h. Proteinase activity was detected as unstained bands on a blue background, representing areas of gelatin digestion. The bands were quantified using Image-J.

### Mitochondrial membrane potential assay

After high-glucose exposure for 6 days, 50 mM of tetramethyl rhodamine methyl ester (TMRM) (Life Technologies, Carlsbad, CA, USA), the active mitochondrial probe was added to the cell culture and further incubated for 1 h at 37°C. Images were collected using an Olympus IX70 inverted epifluorescence microscope (Olympus, Tokyo, Japan), which detects active mitochondria with intense fluorescence (excitation/emission = 549/573 nm). Intensity was calculated using Image-J in a blind manner.

### ATP contents

After high-glucose exposure for 6 days, the cell culture medium was removed from plates, and cells were washed three times with PBS. Lysis buffer (200 mM Tris, pH 7.5, 2 M NaCl, 20 mM EDTA, 1% Triton X-100) was added to lysed cells. ATP Determinant Kit reaction solution (Molecular Probes, Eugene, OR, USA) was added at a volume of 90 µl per well to 10 µl per well of cell lysate. Plates were agitated for 2 min and luminescence was measured.

### Immunostaining

Cells were fixed for 20 min with 4% paraformaldehyde/PBS and permeabilized with 0.2% Triton X-100 for 10 min. After blocking, cells were incubated with primary antibodies; mouse anti-HSP60 antibody (1∶1000 dilution; StressMarq Biosciences) and rabbit anti-CD31 antibody (AnaSpec, San Jose, CA, USA). After washing in PBS, cells were incubated for 1 h at room temperature with fluorescent-linked secondary antibodies (1∶1000 dilution; Pierce Biotechnology). Cells were counterstained for 5 min with Hoechst 33342/PBS solution. The labeled cells were mounted on glass slides and observed under a confocal microscope (FV10i, Olympus, Tokyo, Japan).

### Transmission electron microscopy (TEM)

Cell suspension pellets were fixed with 2.5% glutaraldehyde in 0.1 M phosphate buffer (pH 7.4) at 4°C overnight and then post fixed with 1% osmium tetroxide for 1 h. After dehydration in a series of graded through ethanol, specimens were embedded in Epon 812. Ultrathin sections were cut on a Poster-Blum MT-1 ultra microtome (Ivan Sorvall, Inc., Norwalk, CT, USA), stained with uranyl acetate and lead salts, and examined with a JEM 1010 transmission electron microscope (JEOL, Tokyo, Japan). The decision criterion of abnormal mitochondria were set as collapse of cristae structure or apparent vacuolation, which was quantified in a blinded-manner.

### MitoSOX analysis

The mitochondrial superoxide level was evaluated by MitoSOX Red Mitochondrial Superoxide Indicator, for live-cell imaging which was purchased from Molecular Probes, Inc. (Life Technologies Japan, Osaka, Japan), according to the product Information. After high-glucose exposure for 6 days, EBM-2 medium was removed and 100 µL of 5 µM MitoSOX reagent working solution was applied to cover cells. Incubate cells for 10 minutes at 37°C, protected from light. After incubation, cells were washed gently three times with 100 µL warm PBS. The absorbance at 510/580 nm was measured using a microplate reader.

### Effect of MMP-9 inhibitor on mitochondrial number in high-glucose exposure

In order to confirm that MMP-9 may actually contribute to the mitochondrial defect, we evaluated the effect of selective MMP-9 inhibitor on normal mitochondrial number. After confluence was achieved, the medium was changed to the conditioned medium containing 5.5 mM glucose (control),30 mM glucose(high-glucose, HG), 30 mM glucose plus 100 nM MMP-9 inhibitor, and cultivated for an additional 6 days. During this period, the culture medium was replaced every 2 days. After high-glucose exposure for 6 days, mitochondria was stained by anti HSP60 antibodies as described previously. The number of mitochondria was determinated quantity by using Image-J image-processing software.

### Statistical Analysis

All data are expressed as mean ± SEM. Statistical analysis for the quantitative variable was performed using a one- or two-way ANOVA followed by Student's *t*-test, Dunnett's test, or Tukey's test. The frequencies of death (mortality) were compared by Chi-square test. P<0.05 was considered statistically significant. Analyses were performed using STAT VIEW version 5.0 (SAS Institute, Cary, NC, USA).

## Results

### Body weight, blood glucose levels, and mortality

The *in vivo* experimental protocol is represented in Fig. 1A. At 4 weeks after STZ-treatment, diabetic mice showed siginificantly lower weight and approximately 3 times higher blood glucose levels than control mice (Fig. 1B and C). There was no significant difference in mortality (P = 0.0951) (Fig. 1D).

### Effects of chronic hyperglycemia on hemorrhagic transformation after ischemic stroke

There were no significant differences in infarct area and volume between the groups (Fig. 1E). However, diabetic mice exhibited noticeable aggravation of hemorrhagic transformation in comparison with control mice at 21 h after the reperfusion (Fig. 1F).

### Effects of chronic high-glucose exposure on cell death

The *in vitro* experimental protocol is represented in Fig. 2A. High-glucose exposure for 6 days significantly decreased cell numbers compared with a normal glucose concentration (5.5 mM), as shown by nuclear staining (Fig. 2B). Furthermore, the number of TUNEL-positive cells was markedly increased following high-glucose exposure (Fig. 2C), suggesting that this cell death, at least in part, was due to cellular apoptosis. We then explored the mechanism of this apoptotic cell death. High-glucose exposure significantly increased the activities of caspase-3/7 (Fig. 2D). Of these caspases, immunoblotting analysis indicated that caspase-3, but not caspase-7, activity was significantly increased by high-glucose exposure (Fig. 2E and F). Moreover, we examined the effect of high-glucose exposure on a caspase-independent pathway. The release of both apoptosis inducing factor (AIF) and cytochrome *c* was significantly accelerated by high-glucose exposure. However, no significant difference in nuclear transit of AIF and cytochrome *c* was observed (Fig. 2G). These results suggest that high -glucose exposure may induce endothelial apoptotic cell death through the activation of a caspase-dependent pathway.

### Effects of chronic high-glucose exposure on mitochondrial function

**Figure 3:**
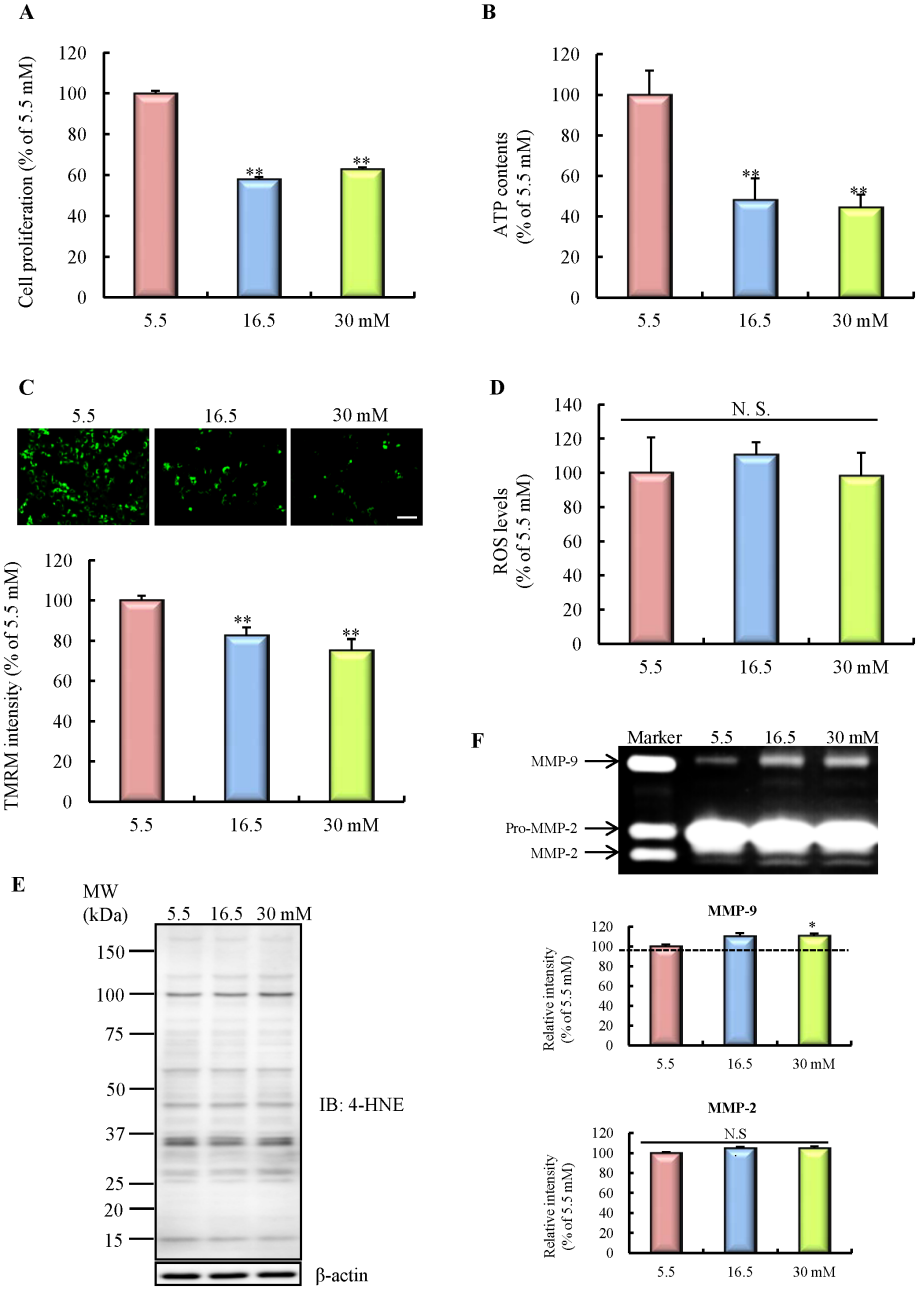
Effects of chronic high-glucose exposure on mitochondrial function in HBMVECs. A: Cell proliferation (n = 7). B: ATP contents (n = 10). C: Mitochondrial membrane potential, determined by tetramethyl rhodamine methyl ester (TMRM) intensity (n = 10). The scale bar indicates 100 µm. D: reactive oxygen species (ROS) levels (n = 10). E: Binding of 4-hydroxy-2-nonenal (4-HNE), an indicator of lipoperoxidation by ROS. F: Enzymatic activities of MMP-2 and MMP-9 by gelatin zymography (n = 3). All data are expressed as mean ± SEM (shown as ratio to 5.5 mM). *P<0.05, **P<0.01 vs. 5.5 mM (Dunnet's test). HBMVECs, human brain microvascular endothelial cells.

Diverse functional assessments were performed to evaluate mitochondrial function under chronic high-glucose exposure. We evaluated three parameters: cell proliferation by the WST-8 assay, ATP contents by the luciferase-based assay, and mitochondrial membrane potential by measuring the TMRM intensity. A significant impairment in all of these functional assessments occurred in high-glucose exposed cells, suggesting that chronic high-glucose exposure may induce mitochondrial dysfunction (Fig. 3A–C). To elucidate the mechanism of this mitochondrial dysfunction, we next examined ROS generation and MMP-2 and MMP-9 activities. We observed that high-glucose exposure did not affect ROS production (Fig. 3D). In agreement with this result, the binding levels of 4-HNE, the major biologically active aldehyde generated from peroxidation of membrane lipids, were not different between the groups (Fig. 3E). In addition, the activity of superoxide dismutase (SOD), which is one of the most important antioxidative enzymes to inactivate the superoxide anion, was not changed by high-glucose exposure (Fig. S1). Moreover, the mitochondrial superoxide level also was not changed by high-glucose exposure (Fig. S3). Alternately, a significant increase of active MMP-9, but not MMP-2, was observed in high-glucose exposed cells (Fig. 3F). Selective MMP-9 inhibitor significantly increased cell proliferation decreased by high-glucose exposure (Fig. S2) and the number of mitochondria was decreased by high-glucose exposure, but this result was suppressed by the administration of an MMP-9 inhibitor (Fig. S4). These data suggest that chronic high-glucose exposure may induce mitochondrial dysfunction, at least in part, via upregulation of MMP-9, in an ROS-independent manner.

### Effects of chronic high-glucose exposure on mitochondrial morphology

**Figure 4:**
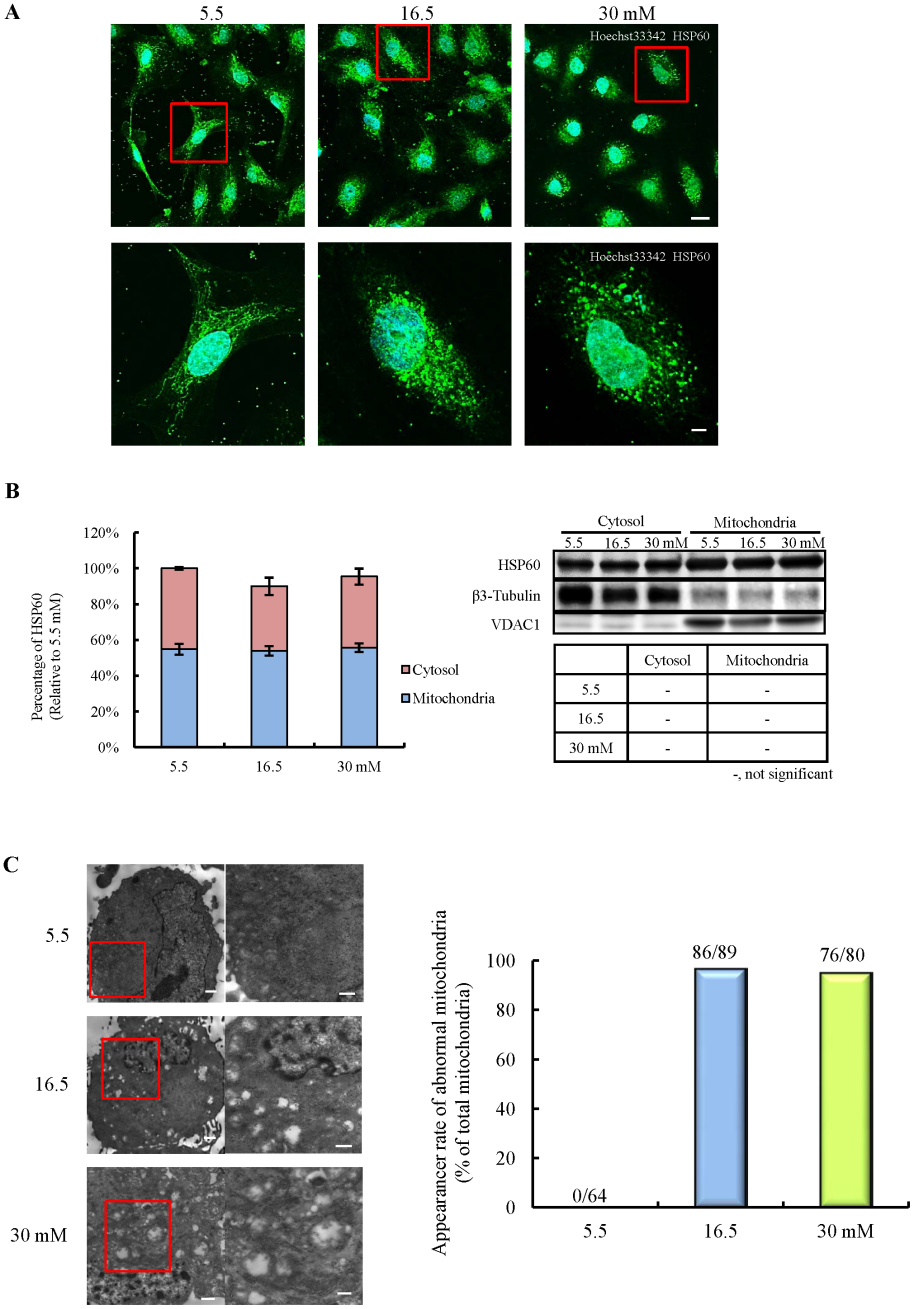
Effects of chronic high-glucose exposure on morphological alteration of mitochondria in HBMVECs. A: Mitochondrial shapes were identified by immunostaining of HSP60, a marker of normal mitochondria. The scale bars indicate 20 µm in upper, and 5 µm in lower pictures, respectively. B: Accumulation of HSP60 in mitochondria, which reflects the quantitative alteration of mitochondria (n = 3). Data are expressed as mean ± SEM. C: More detailed morphological alteration of mitochondria by transmission electron microscopy (TEM). The scale bars indicate 1 µm (left panels) and 500 nm (right panels), respectively. Abnormal mitochondria, described as absolute disintegration of normal mitochondrial structure, were counted (shown as percentage of total mitochondria) (5.5 mM, n = 11; 16.5 mM, n = 15; 30 mM, n = 11).

In an attempt to characterize the mitochondrial changes that occur under chronic high-glucose exposure in more detail, we investigated the morphological alteration of the mitochondria. Immunostaining of HSP60, used as a marker of normal mitochondria, revealed that the mitochondria in high-glucose-exposed cells exhibited apparent shortened forms and fragmentation (Fig. 4A). However, the accumulation of HSP60 in mitochondria was not altered, indicating that the mitochondrial changes were qualitative rather than quantitative (Fig. 4B). We used transmission electron microscopy (TEM) analysis to further characterize the morphological effects of high-glucose exposure. Predictably, high-glucose exposure induced massive fragmentation, vacuolation, and cristae disruption. Furthermore, a marked hypertrophy of mitochondria was observed in high-glucose exposed cells compared with control cells. We also found that endoplasmic reticulum (ER) gathered in close proximity to the mitochondria denatured by high-glucose exposure. In addition, autophagosomal-like structures contained mitochondria were observed in some places. (Fig. 4C). These results suggest that chronic high-glucose exposure induces considerable disturbance of mitochondrial morphology.

### Effects of chronic high-glucose exposure on iCell endothelial cells

**Figure 5:**
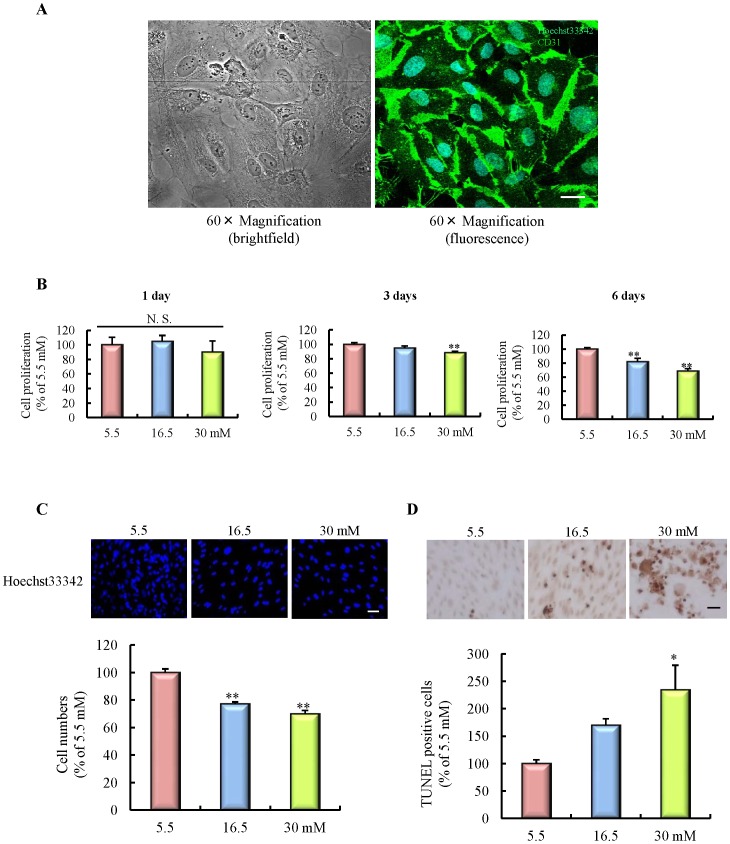
Effects of chronic high-glucose exposure on human endothelial cells derived from iPS cells. A: Characterization of iCell endothelial cells by immunostaining of CD31, a marker of endothelial cells. The scale bar indicates 20 µm. B: Temporal changes of cell proliferation, assessed at 1, 3, and 6 days after the onset of high-glucose exposure (n = 9 or 10). C: Nuclear staining for Hoechst 33342. The number of cells exhibiting nuclear stain was counted (n = 10). The scale bar indicates 50 µm. D: Number of TUNEL-positive cells (n = 4). The scale bar indicates 100 µm. All data are expressed as mean ± SEM (shown as percentage of 5.5 mM). *P<0.05, **P<0.01 vs. 5.5 mM (Tukey's test or Dunnet's test).

We examined whether the phenomena similar to those previously observed would also occur in iPS-induced endothelial cells. Before the experiment, we confirmed that the iPS cells differentiated into normal endothelial cells, by using CD31 immunostaining (Fig. 5A). We measured three parameters: cell proliferation by WST-8 assay, cell numbers by nuclear staining assay, and apoptotic cell death by TUNEL staining. High-glucose exposure significantly decreased cell proliferation and cell numbers, and increased the number of TUNEL-positive cells (Fig. 5B–D). These results were consistent with our previous studies in HBMVECs.

## Discussion

In this study, we used STZ-induced diabetic mice as a model of the chronic complications that occur in human diabetes mellitus. After 4 weeks after STZ administration, the blood glucose level in diabetic mice was 3 times higher than that in control mice. In addition, there was a marked weight loss in diabetic mice along with evident polyposia and polyuria tendency, which closely resemble clinical symptoms of DM. After ischemia/reperfusion injury by middle cerebral artery occlusion (MCAO), infarct area and volume were not altered by diabetes; however, hemorrhagic volume was significantly larger in diabetic mice than in the control mice. This result is consistent with those of previous reports [15], [35], [36], suggesting that chronic hyperglycemia aggravates hemorrhagic transformation of ischemic stroke in mice.

Endothelial dysfunction is considered one of the main contributors of complications in DM; therefore, we next focused on brain microvascular endothelial cells and conducted several experiments to clarify the effects of chronic high-glucose exposure *in vitro*. We established an *in vitro* diabetic model by incubating cells in a high-glucose environment (16.5 or 30 mM) for 6 days because high-glucose exposure at a concentration of 30 mM for 6 days increases endothelial permeability in the brain [37]. In our study, chronic high-glucose exposure resulted in increased apoptotic cell death. Few studies have thus far examined the influence of hyperglycemia on apoptosis in brain microvascular endothelial cells; however high-glucose-induced endothelial apoptosis has been reported in other cell types including human cavernous endothelial cells, human coronary artery endothelial cells and pancreatic islet endothelial cells [38]–[40]. Despite these efforts, no consensus on the underlying mechanisms of apoptotic cell death in DM has been achieved. There are two main apoptosis pathways: caspase-dependent and caspase-independent. We first examined the activities of caspase-3 and -7. High-glucose exposure significantly increased the activity of caspase-3, but not caspase-7. On the other hand, high-glucose exposure promoted the releases of pro-apoptotic peptides, AIF, and cytochrome *c* into the cytosol; however, there was no significant difference in nuclear transit. These results suggest that chronic high-glucose exposure induces apoptotic cell death through the activation of a caspase-dependent pathway in HBMVECs.

Most importantly, we provided novel findings regarding the functional and morphological consequences of altered mitochondrial dynamics under hyperglycemic conditions in HBMVECs. Several previous studies showed that diabetic conditions disrupt mitochondrial integrity in different cell types. For instance, incubation of rat retinal endothelial cells with high-glucose containing medium induces the loss of mitochondrial networks and increases apoptosis [31]. Moreover, exposure of rat hepatocytes to high-glucose induces mitochondrial fragmentation [27]. In the most recent study by Makino et al., endothelial cells isolated from the coronary arteries of diabetic mice displayed fragmented mitochondria and increased ROS production [32]. To the best of our knowledge, this is the first report to investigate the effects of chronic hyperglycemia on the mitochondrial functions and morphological alterations in HBMVECs.

Our results clearly demonstrated that high-glucose exposure impairs mitochondrial functions (decreased cell proliferation, ATP contents, and mitochondrial membrane potential) and induces mitochondrial morphological alterations (massive fragmentation, vacuolation, and cristae disruption). Hyperglycemia induces excess ROS production, including O_2_^−^, in many cell types [41]–[43], leading to cellular apoptosis [42], abnormalities of cell cycling [44], and delayed replication [43]. Interestingly, the present data demonstrated that HBMVECs with hyperglycemia did not exhibit higher production of ROS. Meanwhile, MMP-9 which is a potentially deleterious neurovascular protease [45], associated with blood-brain barrier disruption, neuronal apoptosis and survival [46]–[48], was significantly upregulated under high-glucose exposure. In accordance with this finding, Kumari et al. reported an increase in MMP-9 activity in diabetic db/db mice following hypoxic-cerebral ischemia [47]. In fact, we confirmed that MMP-9 inhibition could partly improve mitochondrial function. Hence, chronic hyperglycemia may exacerbate mitochondrial functions possibly through other deleterious factors, which include, at least in part, MMP-9, rather than via ROS production in the brain. Further studies are required to completely elucidate the mechanisms of hyperglycemia-induced mitochondrial dysfunctions.

Interestingly, we found that many a ER gathered in close proximity to the mitochondria denatured by high-glucose exposure. Furthermore, autophagosomal-like structures contained mitochondria in some places. Therefore, a great deal of attention is currently focused on the relationship between mitochondria-selective autophagy, called “mitophagy” and ER activities [49], [50]. These observations are likely to support the possibility that mitophagy is involved in the pathophysiology of DM via its close relation to ER.

Recently, there has been a growing interest in research or drug development using human iPS cells. In this study, we determined whether similar dysfunctions observed in HBMVECs would also occur in human iPS-induced endothelial cells, and observed several results coincident with these in HBMVECs. The findings strongly indicate that hyperglycemia decreases mitochondrial enzymatic activity and increases apoptotic cell death in brain microvascular endothelial cells.

There are several limitations in the present study. First, *in vivo* MCAO experiments, we used STZ-induced diabetic mice model, which normally induces Type I DM. Clinically most of diabetic patients are classified as Type 2. In regard to this point, there are a little bit differences in animal models. Second, we examined the effect of hyperglycemia on mitochondrial defects only *in vitro* study, thus further investigation is required to clarify whether these results could be truly extrapolated to the results of *in vivo* study. Additionally, *in vitro* experimental design, we only conducted in the chronic high-glucose condition without any ischemic stimuli. Although it makes reasonable to understand the deleterious effect of chronic hyperglycemia on brain endothelial cells, another *in vitro* stroke model (for example, hypoxia or oxygen glucose deprivation) may be also necessary to match the in vivo MCAO experiments.

**Figure 6:**
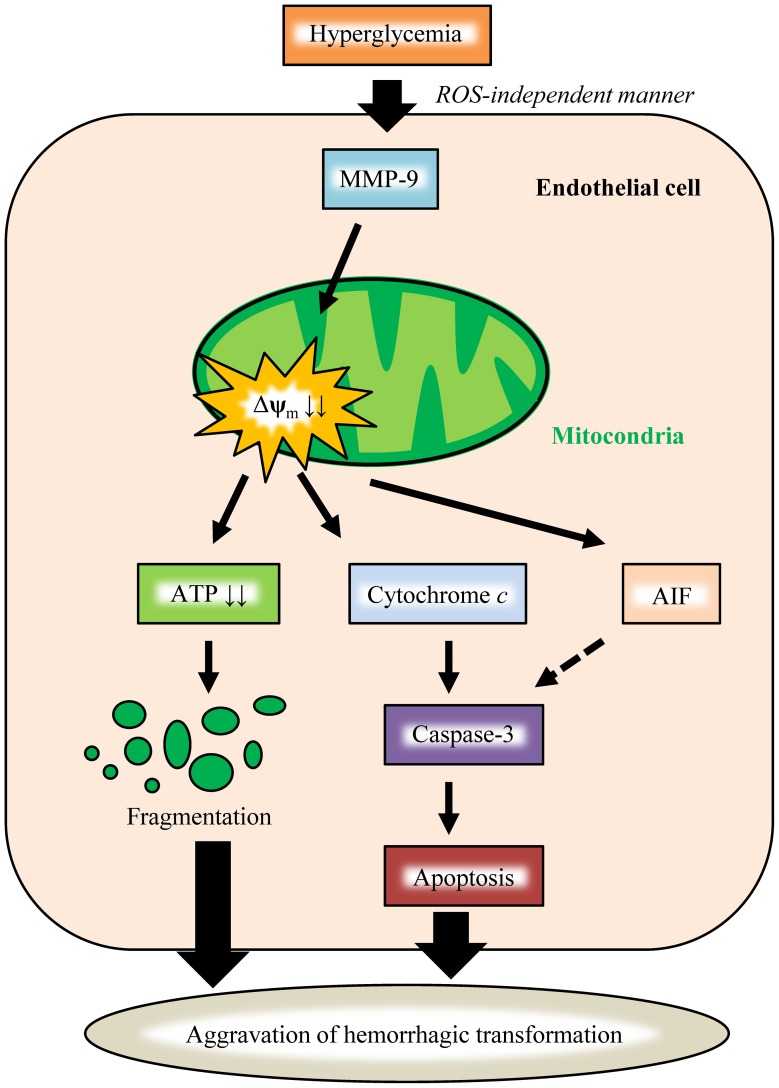
Diagram illustrating the postulated mechanism through which hyperglycemia aggravates hemorrhagic transformation after ischemic stroke. Hyperglycemia increases the activity of MMP-9 in an ROS-independent manner, which promotes the opening of mitochondrial permeability transition pores (mitochondrial depolarization; Δ**ψ**_m_ ↓↓). The mitochondria that have lost normal function can no longer produce ATP, and emit various pro-apoptotic factors, such as AIF and cytochrome c into the cytosol. These factors subsequently activate caspase-3 and induce apoptotic cell death in HBMVECs. On the other hand, functional failure leads to morphological alteration of mitochondria, (fragmentation, vacuolation, cristae disruption). Eventually, both of these functional and morphological disturbances result in the aggravation of hemorrhagic transformation after ischemic stroke.

In summary, chronic hyperglycemia aggravates hemorrhagic transformation after ischemic stroke through mitochondrial dysfunction and morphological alterations, partially via MMP-9 activation with ROS-independent manner; this leads to caspase-dependent apoptotic cell death in endothelial cells (Fig. 6). Hence, mitochondrial protection may lead to the mitigation of complications in DM, and mitochondria-targeting therapy may be a clinically innovative therapeutic strategy for addressing diabetic complications in the future.

## Supporting Information

Figure S1 **Effect of chronic high-glucose exposure on superoxide dismutase (SOD) activity in HBMVECs.** Intracellular SOD activity was assessed in HBMVECs (n = 4, Dunnet's test). The SOD activity was represented as a function of inhibition percentage. All data are expressed as mean ± SEM (shown as inhibition %). HBMVECs, human brain microvascular endothelial cells.
(TIFF)

Figure S2 **Effect of selective MMP-9 inhibitor on cell proliferation decreased by high-glucose exposure in HBMVECs.** Cell proliferation was assessed by Cell Counting Kit-8 in HBMVECs (n = 10). Control means normal glucose concentration at 5.5 mM, and HG means high-glucose concentration at 30 mM. All data are expressed as mean ± SEM (shown as ratio to Control). ^#^P<0.05 vs. Control, *P<0.05 vs. HG (Student's *t*-test). HBMVECs, human brain microvascular endothelial cells.
(TIFF)

Figure S3 **Effect of chronic high-glucose exposure on superoxide level in HBMVECs.** Intracellular superoxide level was assessed in HBMVECs (n = 9–10, Student's *t*-test). The superoxide level was represented as percentage of control. All data are expressed as mean ± SEM (shown % of control). HBMVECs, human brain microvascular endothelial cells.
(TIFF)

Figure S4 **Effect of selective MMP-9 inhibitor on the number of mitochondria by high-glucose exposure in HBMVECs.** The number of mitochondria was identified by immunostaining of HSP60, a marker of normal mitochondria. The scale bars indicate 20 µm. Control (n = 5) means normal glucose concentration at 5.5 mM, and HG (n = 9) means high-glucose concentration at 30 mM. HG+MMP-9 inhibitor (n = 5) means high-glucose concentration at 30 mM with MMP-9 inhibitor. All data are expressed as mean ± SEM (shown as percentage of Control). **P<0.01 vs. Control, ^#^P<0.05 vs. HG (Student's t-test). HBMVECs, human brain microvascular endothelial cells.
(TIFF)

Method S1 **Supplemental methods.**
(DOCX)

## References

[1] Danaei G, Finucane MM, Lu Y, Singh GM, Cowan MJ, et al (2011) National, regional, and global trends in fasting plasma glucose and diabetes prevalence since 1980: systematic analysis of health examination surveys and epidemiological studies with 370 country-years and 2.7 million participants. Lancet 378: 31–40. 21705069 10.1016/S0140-6736(11)60679-X

[2] Wild S, Roglic G, Green A, Sicree R, King H (2004) Global prevalence of diabetes: estimates for the year 2000 and projections for 2030. Diabetes Care 27: 1047–1053. 15111519 10.2337/diacare.27.5.1047

[3] Shaw JE, Sicree RA, Zimmet PZ (2010) Global estimates of the prevalence of diabetes for 2010 and 2030. Diabetes Res Clin Pract 87: 4–14. 19896746 10.1016/j.diabres.2009.10.007

[4] Stegmayr B, Asplund K (1995) Diabetes as a risk factor for stroke. A population perspective. Diabetologia 38: 1061–1068. 8591820 10.1007/BF00402176

[5] Kissela BM, Khoury J, Kleindorfer D, Woo D, Schneider A, et al (2005) Epidemiology of ischemic stroke in patients with diabetes: the greater Cincinnati/Northern Kentucky Stroke Study. Diabetes Care 28: 355–359. 15677792 10.2337/diacare.28.2.355

[6] Stamler J, Vaccaro O, Neaton JD, Wentworth D (1993) Diabetes, other risk factors, and 12-yr cardiovascular mortality for men screened in the Multiple Risk Factor Intervention Trial. Diabetes Care 16: 434–444. 8432214 10.2337/diacare.16.2.434

[7] Folsom AR, Rasmussen ML, Chambless LE, Howard G, Cooper LS, et al (1999) Prospective associations of fasting insulin, body fat distribution, and diabetes with risk of ischemic stroke. The Atherosclerosis Risk in Communities (ARIC) Study Investigators. Diabetes Care 22: 1077–1083. 10388971 10.2337/diacare.22.7.1077

[8] Putaala J, Liebkind R, Gordin D, Thorn LM, Haapaniemi E, et al (2011) Diabetes mellitus and ischemic stroke in the young: clinical features and long-term prognosis. Neurology 76: 1831–1837. 21606455 10.1212/WNL.0b013e31821cccc2

[9] Capes SE, Hunt D, Malmberg K, Pathak P, Gerstein HC (2001) Stress hyperglycemia and prognosis of stroke in nondiabetic and diabetic patients: a systematic overview. Stroke 32: 2426–2432. 11588337 10.1161/hs1001.096194

[10] Pulsinelli WA, Levy DE, Sigsbee B, Scherer P, Plum F (1983) Increased damage after ischemic stroke in patients with hyperglycemia with or without established diabetes mellitus. Am J Med 74: 540–544. 6837584 10.1016/0002-9343(83)91007-0

[11] Martini SR, Kent TA (2007) Hyperglycemia in acute ischemic stroke: a vascular perspective. J Cereb Blood Flow Metab 27: 435–451. 16804552 10.1038/sj.jcbfm.9600355

[12] Kawai N, Keep RF, Betz AL (1997) Hyperglycemia and the vascular effects of cerebral ischemia. Stroke 28: 149–154. 8996504 10.1161/01.str.28.1.149

[13] Quast MJ, Wei J, Huang NC, Brunder DG, Sell SL, et al (1997) Perfusion deficit parallels exacerbation of cerebral ischemia/reperfusion injury in hyperglycemic rats. J Cereb Blood Flow Metab 17: 553–559. 9183293 10.1097/00004647-199705000-00009

[14] Ning R, Chopp M, Yan T, Zacharek A, Zhang C, et al (2012) Tissue plasminogen activator treatment of stroke in type-1 diabetes rats. Neuroscience 222: 326–332. 22820263 10.1016/j.neuroscience.2012.07.018 PMC3474540

[15] Won SJ, Tang XN, Suh SW, Yenari MA, Swanson RA (2011) Hyperglycemia promotes tissue plasminogen activator-induced hemorrhage by Increasing superoxide production. Ann Neurol 70: 583–590. 22002675 10.1002/ana.22538 PMC4554391

[16] Morigi M, Angioletti S, Imberti B, Donadelli R, Micheletti G, et al (1998) Leukocyte-endothelial interaction is augmented by high glucose concentrations and hyperglycemia in a NF-kB-dependent fashion. J Clin Invest 101: 1905–1915. 9576755 10.1172/JCI656 PMC508777

[17] Prakash R, Somanath PR, El-Remessy AB, Kelly-Cobbs A, Stern JE, et al (2012) Enhanced cerebral but not peripheral angiogenesis in the Goto-Kakizaki model of type 2 diabetes involves VEGF and peroxynitrite signaling. Diabetes 61: 1533–1542. 22403298 10.2337/db11-1528 PMC3357273

[18] Hawkins BT, Lundeen TF, Norwood KM, Brooks HL, Egleton RD (2007) Increased blood-brain barrier permeability and altered tight junctions in experimental diabetes in the rat: contribution of hyperglycaemia and matrix metalloproteinases. Diabetologia 50: 202–211. 17143608 10.1007/s00125-006-0485-z

[19] Luan H, Kan Z, Xu Y, Lv C, Jiang W (2013) Rosmarinic acid protects against experimental diabetes with cerebral ischemia: relation to inflammation response. J Neuroinflammation 10: 28. 23414442 10.1186/1742-2094-10-28 PMC3614882

[20] Tureyen K, Bowen K, Liang J, Dempsey RJ, Vemuganti R (2011) Exacerbated brain damage, edema and inflammation in type-2 diabetic mice subjected to focal ischemia. J Neurochem 116: 499–507. 21133923 10.1111/j.1471-4159.2010.07127.x PMC3076322

[21] Smith CJ, Lawrence CB, Rodriguez-Grande B, Kovacs KJ, Pradillo JM, et al (2013) The immune system in stroke: clinical challenges and their translation to experimental research. J Neuroimmune Pharmacol 8: 867–887. 23673977 10.1007/s11481-013-9469-1

[22] McBride HM, Neuspiel M, Wasiak S (2006) Mitochondria: more than just a powerhouse. Curr Biol 16: R551–560. 16860735 10.1016/j.cub.2006.06.054

[23] Contreras L, Drago I, Zampese E, Pozzan T (2010) Mitochondria: the calcium connection. Biochim Biophys Acta 1797: 607–618. 20470749 10.1016/j.bbabio.2010.05.005

[24] Twig G, Hyde B, Shirihai OS (2008) Mitochondrial fusion, fission and autophagy as a quality control axis: the bioenergetic view. Biochim Biophys Acta 1777: 1092–1097. 18519024 10.1016/j.bbabio.2008.05.001 PMC3809017

[25] Molina AJ, Wikstrom JD, Stiles L, Las G, Mohamed H, et al (2009) Mitochondrial networking protects beta-cells from nutrient-induced apoptosis. Diabetes 58: 2303–2315. 19581419 10.2337/db07-1781 PMC2750232

[26] Lowell BB, Shulman GI (2005) Mitochondrial dysfunction and type 2 diabetes. Science 307: 384–387. 15662004 10.1126/science.1104343

[27] Yu T, Robotham JL, Yoon Y (2006) Increased production of reactive oxygen species in hyperglycemic conditions requires dynamic change of mitochondrial morphology. Proc Natl Acad Sci U S A 103: 2653–2658. 16477035 10.1073/pnas.0511154103 PMC1413838

[28] Bach D, Pich S, Soriano FX, Vega N, Baumgartner B, et al (2003) Mitofusin-2 determines mitochondrial network architecture and mitochondrial metabolism. A novel regulatory mechanism altered in obesity. J Biol Chem 278: 17190–17197. 12598526 10.1074/jbc.M212754200

[29] Zorzano A, Liesa M, Palacin M (2009) Role of mitochondrial dynamics proteins in the pathophysiology of obesity and type 2 diabetes. Int J Biochem Cell Biol 41: 1846–1854. 19703653 10.1016/j.biocel.2009.02.004

[30] Widlansky ME, Wang J, Shenouda SM, Hagen TM, Smith AR, et al (2010) Altered mitochondrial membrane potential, mass, and morphology in the mononuclear cells of humans with type 2 diabetes. Transl Res 156: 15–25. 20621033 10.1016/j.trsl.2010.04.001 PMC2904361

[31] Trudeau K, Molina AJ, Guo W, Roy S (2010) High glucose disrupts mitochondrial morphology in retinal endothelial cells: implications for diabetic retinopathy. Am J Pathol 177: 447–455. 20522647 10.2353/ajpath.2010.091029 PMC2893686

[32] Makino A, Scott BT, Dillmann WH (2010) Mitochondrial fragmentation and superoxide anion production in coronary endothelial cells from a mouse model of type 1 diabetes. Diabetologia 53: 1783–1794. 20461356 10.1007/s00125-010-1770-4 PMC2892085

[33] Hara H, Huang PL, Panahian N, Fishman MC, Moskowitz MA (1996) Reduced brain edema and infarction volume in mice lacking the neuronal isoform of nitric oxide synthase after transient MCA occlusion. J Cereb Blood Flow Metab 16: 605–611. 8964799 10.1097/00004647-199607000-00010

[34] Hara H, Friedlander RM, Gagliardini V, Ayata C, Fink K, et al (1997) Inhibition of interleukin 1beta converting enzyme family proteases reduces ischemic and excitotoxic neuronal damage. Proc Natl Acad Sci U S A 94: 2007–2012. 9050895 10.1073/pnas.94.5.2007 PMC20033

[35] Ergul A, Elgebaly MM, Middlemore ML, Li W, Elewa H, et al (2007) Increased hemorrhagic transformation and altered infarct size and localization after experimental stroke in a rat model type 2 diabetes. BMC Neurol 7: 33. 17937795 10.1186/1471-2377-7-33 PMC2098774

[36] Chiu CD, Chen CC, Shen CC, Chin LT, Ma HI, et al (2013) Hyperglycemia exacerbates intracerebral hemorrhage via the downregulation of aquaporin-4: temporal assessment with magnetic resonance imaging. Stroke 44: 1682–1689. 23592763 10.1161/STROKEAHA.113.675983

[37] Yan J, Zhang Z, Shi H (2012) HIF-1 is involved in high glucose-induced paracellular permeability of brain endothelial cells. Cell Mol Life Sci 69: 115–128. 21617913 10.1007/s00018-011-0731-5 PMC11115066

[38] Ning H, Qiu X, Baine L, Lin G, Lue TF, et al (2012) Effects of high glucose on human cavernous endothelial cells. Urology 80: 1162.e1167–1111. 10.1016/j.urology.2012.04.071 22951001

[39] Kageyama S, Yokoo H, Tomita K, Kageyama-Yahara N, Uchimido R, et al (2011) High glucose-induced apoptosis in human coronary artery endothelial cells involves up-regulation of death receptors. Cardiovasc Diabetol 10: 73. 21816064 10.1186/1475-2840-10-73 PMC3161855

[40] Gong L, Liu FQ, Wang J, Wang XP, Hou XG, et al (2011) Hyperglycemia induces apoptosis of pancreatic islet endothelial cells via reactive nitrogen species-mediated Jun N-terminal kinase activation. Biochim Biophys Acta 1813: 1211–1219. 21435358 10.1016/j.bbamcr.2011.03.011

[41] Mukhopadhyay P, Rajesh M, Yoshihiro K, Hasko G, Pacher P (2007) Simple quantitative detection of mitochondrial superoxide production in live cells. Biochem Biophys Res Commun 358: 203–208. 17475217 10.1016/j.bbrc.2007.04.106 PMC2228267

[42] Piconi L, Quagliaro L, Assaloni R, Da Ros R, Maier A, et al (2006) Constant and intermittent high glucose enhances endothelial cell apoptosis through mitochondrial superoxide overproduction. Diabetes Metab Res Rev 22: 198–203. 16453381 10.1002/dmrr.613

[43] Zou MH, Shi C, Cohen RA (2002) High glucose via peroxynitrite causes tyrosine nitration and inactivation of prostacyclin synthase that is associated with thromboxane/prostaglandin H(2) receptor-mediated apoptosis and adhesion molecule expression in cultured human aortic endothelial cells. Diabetes 51: 198–203. 11756341 10.2337/diabetes.51.1.198

[44] Abraham NG, Kushida T, McClung J, Weiss M, Quan S, et al (2003) Heme oxygenase-1 attenuates glucose-mediated cell growth arrest and apoptosis in human microvessel endothelial cells. Circ Res 93: 507–514. 12933701 10.1161/01.RES.0000091828.36599.34

[45] Qiu J, Xu J, Zheng Y, Wei Y, Zhu X, et al (2010) High-mobility group box 1 promotes metalloproteinase-9 upregulation through Toll-like receptor 4 after cerebral ischemia. Stroke 41: 2077–2082. 20671243 10.1161/STROKEAHA.110.590463 PMC3066477

[46] Copin JC, Goodyear MC, Gidday JM, Shah AR, Gascon E, et al (2005) Role of matrix metalloproteinases in apoptosis after transient focal cerebral ischemia in rats and mice. Eur J Neurosci 22: 1597–1608. 16197500 10.1111/j.1460-9568.2005.04367.x

[47] Kumari R, Willing LB, Patel SD, Baskerville KA, Simpson IA (2011) Increased cerebral matrix metalloprotease-9 activity is associated with compromised recovery in the diabetic db/db mouse following a stroke. J Neurochem 119: 1029–1040. 21923664 10.1111/j.1471-4159.2011.07487.x PMC3217107

[48] Murase S, McKay RD (2012) Matrix metalloproteinase-9 regulates survival of neurons in newborn hippocampus. J Biol Chem 287: 12184–12194. 22351756 10.1074/jbc.M111.297671 PMC3320970

[49] Hamasaki M, Furuta N, Matsuda A, Nezu A, Yamamoto A, et al (2013) Autophagosomes form at ER-mitochondria contact sites. Nature 495: 389–393. 23455425 10.1038/nature11910

[50] Saita S, Shirane M, Nakayama KI (2013) Selective escape of proteins from the mitochondria during mitophagy. Nat Commun 4: 1410. 23361001 10.1038/ncomms2400

