# Supplementary figures and images for: Diabetes Mellitus Aggravates Hemorrhagic Transformation after Ischemic Stroke via Mitochondrial Defects Leading to Endothelial Apoptosis
Source: PLoS One. 2014 Aug 18;9(8):e103818. doi: 10.1371/journal.pone.0103818 (PMC4136737; doi:10.1371/journal.pone.0103818)

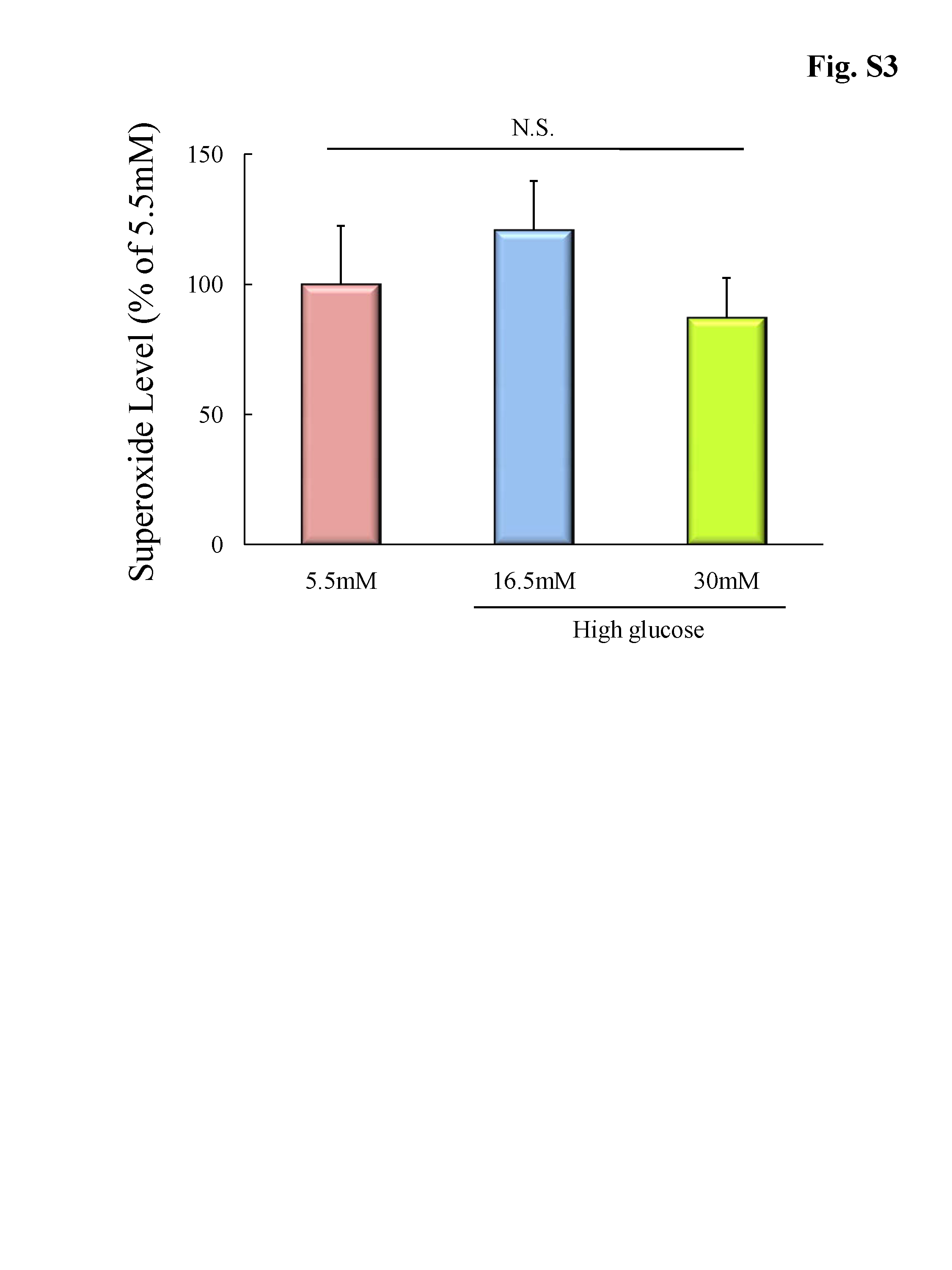

Supplement: Figure S3 — Effect of chronic high-glucose exposure on superoxide level in HBMVECs. Intracellular superoxide level was assessed in HBMVECs (n = 9–10, Student's t-test). The superoxide level was represented as percentage of control. All data are expressed as mean ± SEM (shown % of control). HBMVECs, human brain microvascular endothelial cells. (TIFF) [file pone.0103818.s003.tiff]

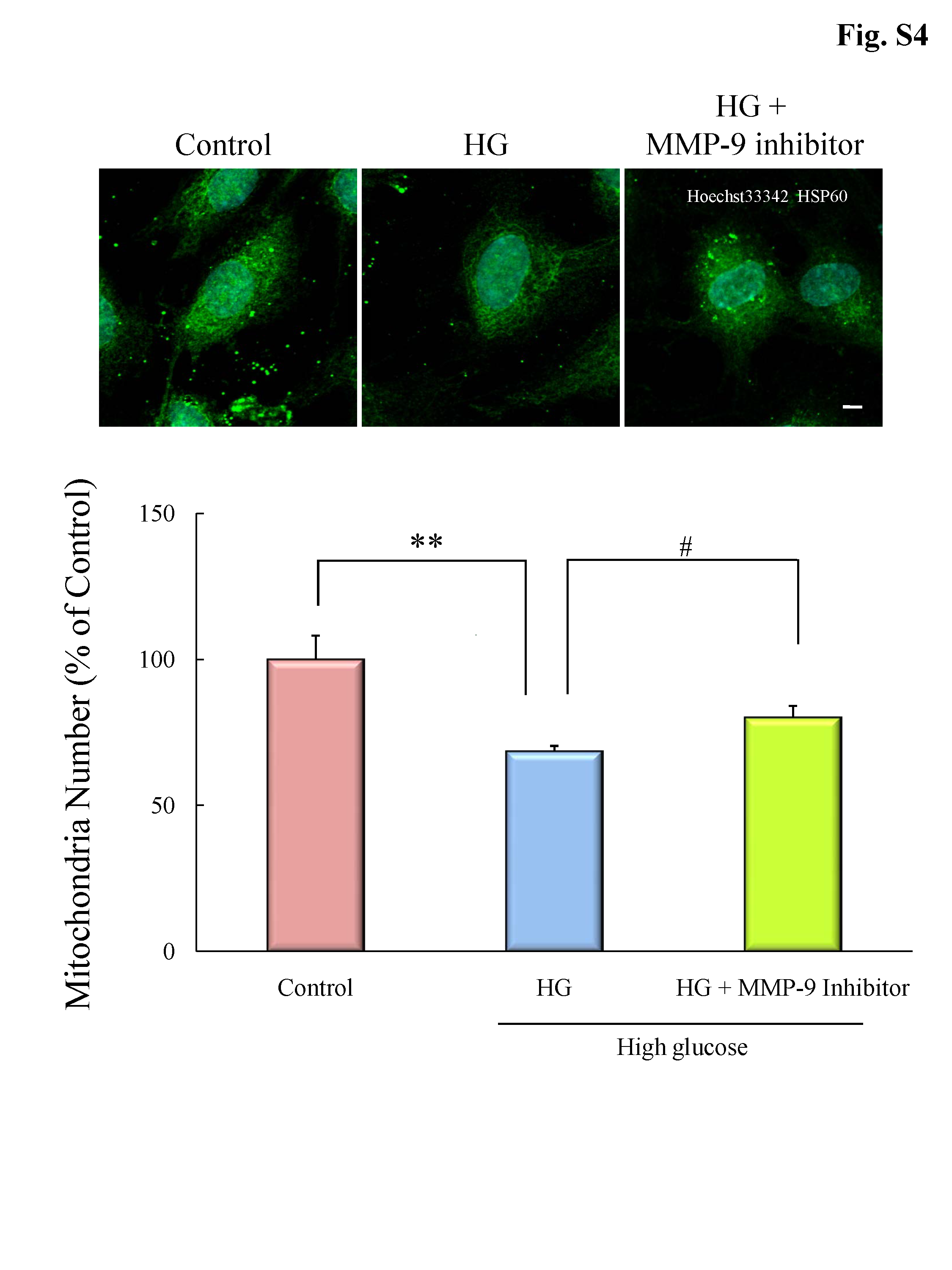

Supplement: Figure S4 — Effect of selective MMP-9 inhibitor on the number of mitochondria by high-glucose exposure in HBMVECs. The number of mitochondria was identified by immunostaining of HSP60, a marker of normal mitochondria. The scale bars indicate 20 µm. Control (n = 5) means normal glucose concentration at 5.5 mM, and HG (n = 9) means high-glucose concentration at 30 mM. HG+MMP-9 inhibitor (n = 5) means high-glucose concentration at 30 mM with MMP-9 inhibitor. All data are expressed as mean ± SEM (shown as percentage of Control). **P<0.01 vs. Control, #P<0.05 vs. HG (Student's t-test). HBMVECs, human brain microvascular endothelial cells. (TIFF) [file pone.0103818.s004.tiff]
