# Supplementary material for: Diabetes Mellitus Aggravates Hemorrhagic Transformation after Ischemic Stroke via Mitochondrial Defects Leading to Endothelial Apoptosis
Source: PLoS One. 2014 Aug 18;9(8):e103818. doi: 10.1371/journal.pone.0103818 (PMC4136737; doi:10.1371/journal.pone.0103818)
Supplement: Method S1 — Supplemental methods. (DOCX) [file pone.0103818.s005.docx]

**Text S1**

*Measurement of Superoxide Dismutase (SOD)*

Intracellular SOD was evaluated using OxiSelect Superoxide Dismutase Activity Assay Kit (Cell Biolabs, San Diego, CA, USA) , according to the manufacturer's instructions.After high-glucose exposure for 6 days, cells were washed with 10 mL ice-cold PBS per 100 mm dish. Then, cells were lysed with 1 mL of cold 1×Lysis Buffer (10 mM Tris, pH 7.5, 150 mMNaCl, 0.1 mM EDTA). The cell lysate was centrifuged at 12000 g for 10 min and collected the supernatant as a sample. Each sample was added to reaction reagent which prepared just prior to use in 96-well microtiter plate. Finally, 10 µL of pre-diluted 1×xanthine oxidase solution was added to each well and incubated for 1 h 37°C. The absorbance at 490 nm was measured using a microplate reader (Varioskan Flash 2.4; Thermo Fisher Scientific).

*Inhibition of MMP-9 activity*

In order to confirm that MMP-9 may actually contribute to the mitochondrial dysfunction, we evaluated the effect of selective MMP-9 inhibitor on cell proliferation as an index of mitochondrial functions. After confluence was achieved, the medium was changed to the conditioned medium containing 5.5 mMglucose (control),30 mM glucose(high-glucose, HG), 30 mMglucose plus 100 nMMMP-9 inhibitor (Millipore, Tokyo, Japan), and cultivated for an additional 6 days. During this period, the culture medium was replaced every 2 days. Cell proliferation was assessed as described previously.

*MitoSOX analysis*

The mitochondrial superoxide level was evaluated by MitoSOX Red Mitochondrial Superoxide Indicator, for live-cell imaging which was purchased from Molecular Probes, Inc. (Life Technologies Japan, Osaka, Japan), according to the product Information. After high-glucose exposure for 6 days, EBM-2 medium was removed and 100 µL of 5 µM MitoSOX™ reagent working solution was applied to cover cells. Incubate cells for 10 minutes at 37°C, protected from light. After incubation, cells were washed gently three times with 100 µL warm PBS. The absorbance at 510/580 nm was measured using a microplate reader.

*Effect of MMP-9 inhibitor on mitochondrial number in high-glucose exposure*

In order to confirm that MMP-9 may actually contribute to the mitochondrial defect, we evaluated the effect of selective MMP-9 inhibitor on normal mitochondrial number. After confluence was achieved, the medium was changed to the conditioned medium containing 5.5 mM glucose (control),30 mM glucose(high-glucose, HG), 30 mM glucose plus 100 nM MMP-9 inhibitor, and cultivated for an additional 6 days. During this period, the culture medium was replaced every 2 days. After high-glucose exposure for 6 days, mitochondria was stained by anti HSP60 antibodies as described previously. The number of mitochondria was determinated quantity by using Image-J image-processing software.

**Supplemental Figure Legends**

**Figure S1. Effect of chronic high-glucose exposure on superoxide dismutase (SOD) activity in HBMVECs.**

Intracellular SOD activity was assessed in HBMVECs (n = 4, Dunnet’s test). The SOD activity was represented as a function of inhibition percentage. All data are expressed as mean ± SEM (shown as inhibition %). HBMVECs, human brain microvascular endothelial cells.

**Figure S2. Effect of selective MMP-9 inhibitor on cell proliferation decreased by high-glucose exposure in HBMVECs.**

Cell proliferation was assessed by Cell Counting Kit-8 in HBMVECs (n = 10). Control means normal glucose concentration at 5.5 mM, and HG means high-glucose concentration at 30 mM. All data are expressed as mean ± SEM (shown as ratio to Control). ^#^P < 0.05 vs. Control, *P < 0.05 vs. HG (Student’s *t*-test). HBMVECs, human brain microvascular endothelial cells.

**Figure S3. Effect of chronic high-glucose exposure on superoxide level in HBMVECs.**

Intracellular superoxide level was assessed in HBMVECs (n = 9 - 10, Student’s *t*-test). The superoxide level was represented as percentage of control. All data are expressed as mean ± SEM (shown % of control). HBMVECs, human brain microvascular endothelial cells.

**Figure S4. Effect of selective MMP-9 inhibitor on the number of mitochondria by high-glucose exposure in HBMVECs.**

The number of mitochondria was identified by immunostaining of HSP60, a marker of normal mitochondria. The scale bars indicate 20 µm. Control (n = 5) means normal glucose concentration at 5.5 mM, and HG (n = 9) means high-glucose concentration at 30 mM. HG + MMP-9 inhibitor (n = 5) means high-glucose concentration at 30 mM with MMP-9 inhibitor. All data are expressed as mean ± SEM (shown as percentage of Control). **P < 0.01 vs. Control, ^#^P < 0.05 vs. HG (Student’s t-test). HBMVECs, human brain microvascular endothelial cells.
